# Metabolites Potentially Derived from Gut Microbiota Associated with Podocyte, Proximal Tubule, and Renal and Cerebrovascular Endothelial Damage in Early Diabetic Kidney Disease in T2DM Patients

**DOI:** 10.3390/metabo13080893

**Published:** 2023-07-28

**Authors:** Lavinia Balint, Carmen Socaciu, Andreea Iulia Socaciu, Adrian Vlad, Florica Gadalean, Flaviu Bob, Oana Milas, Octavian Marius Cretu, Anca Suteanu-Simulescu, Mihaela Glavan, Silvia Ienciu, Maria Mogos, Dragos Catalin Jianu, Sorin Ursoniu, Victor Dumitrascu, Daliborca Vlad, Roxana Popescu, Ligia Petrica

**Affiliations:** 1 Department of Internal Medicine II—Division of Nephrology, “Victor Babes” University of Medicine and Pharmacy, Eftimie Murgu Sq. No. 2, County Emergency Hospital Timisoara, 300041 Timisoara, Romania; lavinia.balint@umft.ro (L.B.); bob.flaviu@umft.ro (F.B.); milas.oana@umft.ro (O.M.); anca.simulescu@umft.ro (A.S.-S.); patruica.mihaela@umft.ro (M.G.); ienciu_silviaoana@yahoo.com (S.I.); maria.stefan2014@yahoo.com (M.M.); petrica.ligia@umft.ro (L.P.); 2 Centre for Molecular Research in Nephrology and Vascular Disease, Faculty of Medicine, “Victor Babes” University of Medicine and Pharmacy, Eftimie Murgu Sq. No. 2, 300041 Timisoara, Romania; carmen.socaciu@usamvcluj.ro (C.S.); vlad.adrian@umft.ro (A.V.); jianu.dragos@umft.ro (D.C.J.); sursoniu@umft.ro (S.U.); dumitrascu.victor@umft.ro (V.D.); vlad.daliborca@umft.ro (D.V.); popescu.roxana@umft.ro (R.P.); 3 Research Center for Applied Biotechnology and Molecular Therapy Biodiatech, SC Proplanta, Trifoiului 12G, 400478 Cluj-Napoca, Romania; 4 Department of Occupational Health, University of Medicine and Pharmacy “Iuliu Haţieganu”, Victor Babes 8, 400347 Cluj-Napoca, Romania; andreeaiso@gmail.com; 5 Department of Internal Medicine II—Division of Diabetes and Metabolic Diseases, “Victor Babes” University of Medicine and Pharmacy, Eftimie Murgu Sq. No. 2, County Emergency Hospital Timisoara, 300041 Timisoara, Romania; 6 Department of Surgery I—Division of Surgical Semiology I, “Victor Babes” University of Medicine and Pharmacy, Eftimie Murgu Sq. No. 2, Emergency Clinical Municipal Hospital Timisoara, 300041 Timisoara, Romania; tavicretu@yahoo.com; 7 Department of Neurosciences—Division of Neurology, “Victor Babes” University of Medicine and Pharmacy, Eftimie Murgu Sq. No. 2, County Emergency Hospital Timisoara, 300041 Timisoara, Romania; 8 Centre for Cognitive Research in Neuropsychiatric Pathology (Neuropsy-Cog), Faculty of Medicine, “Victor Babes” University of Medicine and Pharmacy, Eftimie Murgu Sq. No. 2, 300041 Timisoara, Romania; 9 Department of Functional Sciences III, Division of Public Health and History of Medicine, “Victor Babes” University of Medicine and Pharmacy, Eftimie Murgu Sq. No. 2, 300041 Timisoara, Romania; 10 Centre for Translational Research and Systems Medicine, Faculty of Medicine, “Victor Babes” University of Medicine and Pharmacy, Eftimie, Murgu Sq. No. 2, 300041 Timisoara, Romania; 11 Department of Biochemistry and Pharmacology IV, Division of Pharmacology, “Victor Babes” University of Medicine and Pharmacy, No. 2, Eftimie Murgu Sq., 300041 Timisoara, Romania; 12 Department of Microscopic Morphology II, Division of Cell and Molecular Biology II, “Victor Babes” University of Medicine and Pharmacy, No. 2, Eftimie Murgu Sq., 300041 Timisoara, Romania

**Keywords:** early DKD biomarkers, CSVD, metabolites, arginine, butenoylcarnitine, indoxyl sulfate, p-cresyl sulfate, sorbitol

## Abstract

Complications due to type 2 diabetes mellitus (T2DM) such as diabetic kidney disease (DKD) and cerebral small vessel disease (CSVD) have a powerful impact on mortality and morbidity. Our current diagnostic markers have become outdated as T2DM-related complications continue to develop. The aim of the investigation was to point out the relationship between previously selected metabolites which are potentially derived from gut microbiota and indicators of endothelial, proximal tubule (PT), and podocyte dysfunction, and neurosonological indices. The study participants were 20 healthy controls and 90 T2DM patients divided into three stages: normoalbuminuria, microalbuminuria, and macroalbuminuria. Serum and urine metabolites were determined by untargeted and targeted metabolomic techniques. The markers of endothelial, PT and podocyte dysfunction were assessed by ELISA technique, and the neurosonological indices were provided by an ultrasound device with high resolution (MYLAB 8-ESAOTE Italy). The descriptive statistical analysis was followed by univariable and multivariable linear regression analyses. In conclusion, in serum, arginine (sArg), butenoylcarnitine (sBCA), and indoxyl sulfate (sIS) expressed a biomarker potential in terms of renal endothelial dysfunction and carotid atherosclerosis, whereas sorbitol (sSorb) may be a potential biomarker of blood–brain barrier (BBB) dysfunction. In urine, BCA and IS were associated with markers of podocyte damage, whereas PCS correlated with markers of PT dysfunction.

## 1. Introduction

Diabetes mellitus (DM) is a disease which has a great impact on quality of life and on the global economy by being the determinant of various complications. Type 2 diabetes mellitus (T2DM) is far more frequent than type 1 diabetes mellitus (T1DM), being encountered in 90–95% of all cases of DM. The main targeted sites of T2DM complications are the central and peripheral nervous systems, the kidney, and the eye [1]. On the other hand, gut dysbiosis resulting from unhealthy dietary habits, with subsequent obesity and insulin resistance, is gaining more and more attention concerning its involvement in DM development. The dysregulated gut homeostasis is translated by low butyrate-producing bacteria and the subsequent enhanced oxidative stress [2].

The endothelial cell is hypersensitive to oxidative stress and to high glucose levels, these together leading to its dysfunction. Thus, endothelial dysfunction is defined by impaired vasodilatation through low levels of nitric oxide (NO), a pro-coagulant intravascular state, and massive release of vasoactive agents such as adhesion molecules, chemokines and cytokines, a process that is at the root of diabetes complications [3].

Although they are located in different organs, the blood–brain barrier (BBB) and the glomerular filtration barrier share similar features (endothelial cells, basement membranes and highly differentiated, either brain-specific cells (pericytes and astrocytes) or kidney-specific cells (podocytes)). These structures are very sensitive to the aggression of the hyperglycemic milieu and are very vulnerable to the activation of the immune system that evolves together with a smoldering pro-inflammatory status, a process that starts before the diagnosis of T2DM becomes clinically overt [1,4,5]. Hence, the diagnosis of diabetic kidney disease (DKD) and cerebral small vessel disease (CSVD) are made long after their commencement.

The increased incidence of DKD suggests that our current, traditional biomarkers (eGRF, the estimated glomerular filtration rate, and uACR, the urinary albumin-to-creatinine ratio) are not enough, and fail to be indicators of incipient disease. In our contemporary times, multiple studies have developed with a focus on early biomarkers of podocyte, tubular, and endothelial damage. With regard to podocyte damage, it was demonstrated that urinary synaptopodin correlates with serum creatinine and is a marker of podocyte injury, regardless of albuminuria [6]. In parallel, podocalyxin, another protein responsible for maintaining podocyte cytoskeleton and slit pore diaphragm integrity, is highly expressed in urine in the context of podocyte damage [7]. By shifting the focus to PT damage, Coca et al. discovered an over-expression of a protein found in the apical membranes of proximal tubular epithelial cells, namely the kidney injury molecule 1 (KIM-1), as a response to certain injuries [8]. Proximal tubular cells also express N-acetyl-β-D-glucosaminidase (NAG) which was recognized to be a marker of tubular injury, along with KIM-1 [9].

Current methods of CSVD diagnosis, related to T2DM, mostly consist of magnetic resonance imaging (MRI), which may reveal white matter hyperintensities, polytopic microinfarcts, cerebral atrophy etc. [10]; extracranial Doppler ultrasound of carotid arteries which may reveal atherosclerosis; and transcranial Doppler ultrasound, which provides certain indices that reflect arteriosclerosis and microvasculature dilatation capacity [11].

Ultra-modern techniques of biomarker discovery have been developed recently, such as metabolomics, a method which permits the identification of multiple metabolites belonging to certain metabolic pathways, offering a wider perspective of the functionality of these pathways. In our previous untargeted multivariate and univariate study, it has come to our attention that nitrogen metabolic pathway is deeply involved in early DKD pathogenesis, notably through the phenylalanine, tyrosine, and tryptophan pathways [12]. Going further, in a second study, we have managed to point out an implication of gut-derived metabolites belonging to these pathways in early DKD, by quantitative analysis, independent of albuminuria levels. We have also explored additional metabolites related to mitochondrial dysfunction belonging to the acylcarnitine pathway [13].

Our current research interest focusses on the involvement of targeted metabolites (arginine (Arg), hippuric acid (HA), indoxyl sulfate (IS), p-cresyl sulfate (PCS), L-acetylcarnitine (LAC), butenoylcarnitine (BCA), and sorbitol (Sorb)) at podocyte, tubule, and endothelial level.

This study aims to demonstrate the possible inter-connection of gut-derived metabolites (previously determined through untargeted ultra-high-performance liquid chromatography coupled with electrospray-ionization quadrupole time-of-flight mass spectrometry (UHPLC-QTOF-ESI+-MS) and targeted techniques performed with pure standards) with podocyte, renal proximal tubule, and with endothelial dysfunction biomarkers. Moreover, this study has in view the evaluation of a potential association of the above-mentioned metabolites with early cerebral vessel remodeling in T2DM patients.

## 2. Materials and Methods

### 2.1. Selection of Participants for the Study, and Ethical Standards

Between July 2021 and April 2022, the screening was performed of 130 patients with T2DM admitted to the Nephrology Department and to the Diabetes and Metabolic Diseases Department, from the County Emergency Hospital, Timisoara, Romania. The study was designed to be a pilot and cross-sectional study. It consisted of the enrollment, from the general physician’s office, of 90 T2DM patients (P group) and of 20 healthy individuals (C group) [12,13]. UACR was the parameter by which the pathological group (P) was divided into 3 subgroups (P1—normal to mildly increased albuminuria/normoalbuminuria, P2—moderately increased albuminuria/microalbuminuria, and P3—severely increased albuminuria/macroalbuminuria), based on the KDIGO and ADA Consensus [14].The patients included had been suffering from T2DM for at least 5 years, had no clinical symptoms or history of cerebrovascular disease, and their HbA1c did not exceed 10%. Patients with an HbA1c of over 10%, with other glomerular nephropathies, active infections (especially urinary tract infections), cancers, autoimmune, and psychiatric diseases, were not included in the study. All DKD patients were treated with oral antidiabetic agents and/or insulin, angiotensin 2-converting enzyme inhibitors/angiotensin 2 receptor blockers, and statins.

This research study was authorized by the Ethics Committees of the Victor Babes University of Medicine and Pharmacy, Timisoara (29/30.06.2021) and the County Emergency Hospital Timisoara (220/18.01.2021). Written informed consent was given by all participants, and the study procedures were guided according to Declaration of Helsinki criteria.

### 2.2. The Preparation of Samples

Blood was collected by venipuncture in sterile vacutainers without anticoagulant, and sterile vials were used in order to collect urine samples. Sample preparation for UHPLC-QTOF-ESI+-MS analysis was performed as follows: for each 0.2 mL of serum or urine, a volume of 0.8 mL mix of pure HPLC-grade Methanol and Acetonitrile (2:1 *v/v*) was added. For each type of sample, the mixture was ultrasonicated for 5 min, vortexed and then kept for 24 h at −20 °C for protein precipitation. The supernatant was later prepared by centrifugation, filtered using Nylon filters of 0.2 μm, placed in micro vials and then put into the autosampler of the ultra-high-performance liquid chromatograph (UHPLC) before injection. Subsequently, the supernatant was moved into an autosampler vial for HPLC-MS analysis. The quality control (QC) that consisted of mixing each serum and each urine sample was also carried out, this mix being injected at the beginning, at the end and as every 10th injection, during the analysis of the study samples.

All the specimens were stored at −80 °C prior to analysis. Their collection was performed in triplicate for each subject, and they were labeled using classified numerical codes and evaluated in agreement with the indications given by the manufacturer. Ultimately, the samples were used for UHPLC-QTOF-ESI+-MS analysis and the enzyme-linked immunosorbent assay (ELISA).

### 2.3. Analytical Methods

#### 2.3.1. UHPLC-QTOF-ESI+-MS Analysis

The metabolomic profiling was performed by UHPLC-QTOF-ESI+-MS, using a ThermoFisher Scientific UHPLC Ultimate 3000 instrument equipped with a quaternary pump, Dionex delivery system, and MS detection equipment with MaXis Impact (Bruker Daltonics, Billerica, MA, USA). Detailed information about the methods used for untargeted UHPLC-QTOF-ESI+-MS analysis can be found, in an extended version, in our first study [12].

In order to quantify the metabolites, the reagents and the chemicals that were utilized were the following: PLC-grade formic acid from Sigma-Aldrich (Burlington, MA, USA) and HPLC/MS-grade formic acid and acetonitrile from Fisher Scientific (Pittsburg, PA, USA). The pure standard biomarkers that were utilized were represented by acetyl-L-carnitine hydrochloride (J6153606; Alfa Aesar by Thermo Fisher) MW = 203; arginine from Amino acid standard H (product #20088, Thermo Scientific) MW = 174; asymmetric dimethyl-L-arginine ≥ 95% (HPLC) CAS (Thermo Scientific) 30315-93-6 (Sigma Aldrich) MW = 202.25; hippuric acid, 98%, (A1269022; Alfa Aesar by Thermo Fisher) MW = 179; indoxyl sulfate potassium salt, 97%, (A1707901; Alfa Aesar by Thermo Fisher) MW = 213; Sorbitol, >98% product S1876 Sigma-Aldrich Chemie GmbH, MW = 182; p-Cresyl sulfate, >98%, product 29,504 Cayman Chemical, US, MW = 188, and creatinine > 98% product C4255, Sigma-Aldrich Chemie GmbH, MW = 113. As an internal standard, Doxorubicin hydrocholride (MW = 580) (injectable, 2 mg/mL Sun Pharmaceutical Industries) was used. The ultra-high-purity water was prepared by Millipore-Q Water Purification System (Millipore, Germany). LC–MS grade MeOH, MeCN, and formic acid were purchased from Fisher Scientific (Loughborough, UK). Ultra-pure water was purified using a Milli-Q water system (Millipore, Milford, MA, USA). Instruments used in this study included a vortex mixer, Minicentrifuge Eppendorf (Thermo Fisher Scientific, USA), UPLC-Q-TOF/MS (Bruker GmbH, Bremen, Germany).

#### 2.3.2. ELISA Technique

The ELISA technique was utilized in order to assess the biomarkers of endothelial dysfunction, such as monocyte chemotactic protein-1 ((MCP-1)-Catalogue Nr. E-EL-H6005 Elabscience; sensitivity−37.5 pg/mL, detection range−62.5–4000 pg/mL, coefficient of variance (CV) < 10%) and intercellular adhesion molecule-1 ((ICAM-1)- (Catalogue Nr. E-EL-H6114 Elabscience; sensitivity−0.19 ng/mL, detection range−0.31–20 ng/mL, CV < 10%)); biomarkers of podocyte injury, such as synaptopodin (Catalogue Nr. abx055120 Abbexa; sensitivity—0.10 ng/mL; detection range—0.156–10 ng/mL; coefficient of variance (CV) < 10%) and podocalyxin (Catalogue Nr. E-EL-H2360 Elabscience, sensitivity—0.1 ng/mL; detection range—0.16–10 ng/mL; coefficient of variance (CV) < 10%); and of proximal tubule dysfunction, such as KIM-1 (Catalogue Nr. E-EL-H6029 Elabscience; sensitivity—4.69 pg/mL; detection range—7.81–500 pg/mL; CV < 10%) and NAG (Catalogue Nr. E-EL-H0898 Elabscience; sensitivity—0.94 ng/mL; detection range—1.56–100 ng/mL; CV < 10%).

#### 2.3.3. Cerebrovascular Ultrasound Assessments

The cerebrovascular indices were assessed by an ultrasound device with high resolution (MYLAB 8—ESAOTE Italy), equipped with a Color Ultrasound System that included 2 transducers: one with a selectable minimum frequency band between 1.7 MHz and 4 MHz (multifrequency sectorial transducer—phased array), and the other one with a frequency from 3.6 MHz to 16 MHz (linear transducer).

The evaluation of intima-media thickness (IMT) was accomplished bilaterally, in the common carotid arteries (CCA). This reflects the distance between the luminal–intimal and media–adventitial interfaces of the common carotid arteries, and is represented by a double-line pattern displayed by ultrasound in brightness mode (B-mode), in a longitudinal plane. The normal cut-off points were set below 1.0 mm. Three evaluations of IMT for each subject were performed, and the highest value was taken into consideration.

The pulsatility indices (PI) and resistance indices (RI) were evaluated in both internal carotid arteries (ICAs), with a continuous wave of 4 MHz-CW, by extracranial Doppler ultrasound, and in both middle cerebral arteries (MCAs), with a pulsed wave of 2 MHz-PW, by transcranial Doppler ultrasound. The Gosling’s PI was calculated automatically using the following formula: (sytolic flow velocity—diastolic flow velocity)/mean flow velocity) with a cut-off point set at <1. The Pourcellot’s RI was calculated using the (systolic flow velocity—diastolic flow velocity)/systolic flow velocity) formula, with a normal value below 0.7. This measurement provided data about the arteries examined (ICAs and MCAs), which are commonly known as low-resistance vessels. Thus, increased PI and RI in the ICAs and MCAs indicate increased resistance or the loss of vasodilatation capability of these vessels.

The breath-holding test (BHT) was performed in order to measure the cerebrovascular reactivity (CVR) in the MCAs, bilaterally, with the same equipment as mentioned before. This technique is performed after normal breathing for 4 min, followed by holding the breath after a normal inspiration. In all this time, the blood mean flow velocity (MFV) and the time of breath-holding were recorded continuously. The maneuver is repeated after 2 or 3 min of rest, to permit the MFVs to return to normal. After repeating the procedure, the mean MCA-MFV values and mean breath-holding times are calculated. The breath-holding index (BHI) is calculated as percent increase in MCA- MFV recorded by breath-holding divided by seconds of breath-holding [(Vbh − Vr/Vr)·100·s^−1^], where Vbh is MCA-MFV at the end of breath-holding, Vr the MCA-MFV at rest, and s^−1^ per second of breath-holding. The CVR is defined by the auto-regulatory vasodilatation of cerebral vessels as a response to a vasodilator trigger such as hypercapnia. Normal ranges for BHI were set between 1.2 ± 0.6, whereas a BHI under 0.5 was considered pathological.

### 2.4. The Integration of the Results and Statistical Analysis

A first study of the same samples was performed, and it consisted of untargeted multivariate and univariate metabolomic analyses. Firstly, untargeted multivariate analysis was performed in order to discriminate the metabolites between group P and group C, Additional statistical assessments were used, such as fold change, principal component analysis (PCA) and partial least squares discriminant analysis (PLSDA) score plots, including VIP values. Log2 fold change values and Bonferroni-adjusted *p*-values were used for the generation of volcano plots.

Secondly, through univariate analysis, one-way ANOVA allowed for the discrimination of metabolites between subgroups (C, P1, P2 and P3). Additionally, a large amount of data were obtained by categorizing these metabolites into classes. The main classes of metabolites and derivatives included organic and free amino acids, tryptophan metabolites, uremic toxins, carnitines, antioxidants and cell regulators, and carbohydrate metabolites. Subsequently, the metabolic pathways were investigated to identify the specific metabolites that distinguished the normoalbuminuric group from the other groups. In this step, hypotheses regarding the role of metabolites in early DKD were emitted. Based on these hypotheses, metabolites from the nitrogen metabolic pathway were chosen for additional targeted analysis [12].

In our second study, the metabolites that showed biomarker potential and were proven to be derived from gut microbiota based on the literature data, were quantified in the plasma and urine. At that point, by observing the dynamics of peak intensities of acylcarnitine and carbohydrate metabolites in the normoalbuminuric category, it was decided to also include the metabolites derived from these pathways for quantification. Information about stock solutions and the validation methods can be found in detail in our second study [13].

In the first two studies, we examined and discussed the dynamics of metabolic pathways and their resulting metabolites, the correlation between serum and urine findings, and their influence on the physiopathological mechanisms of early DKD [12,13].

## 3. Results

### 3.1. Clinical Features, Biological Results, and Neurosonological Indices

Table 1 presents the data resulting from history and the clinical examination, the common biological parameters, and the ones resulting from the ELISA assay and UHPLC-QTOF-ESI+-MS techniques, as well as the neurosonological indices. The data are presented as means ± standard deviations (SD). The comparison between controls (C) and stages of DKD was realized by one-way ANOVA with Bonferroni correction, a chi-squared test and the Kruskal–Wallis test, whereas the comparison between subgroups, namely C vs. P1, P1 vs. P2 and P2 vs. P3, was carried out by applying Student’s *t*-test, the chi-squared test and the Mann–Whitney test.

Furthermore, after performing the aforementioned descriptive statistical analysis, it was revealed that decreased levels of sArg and increased levels of sIS, sBCA, and sSorb differentiate between C and DKD subgroups and P1 vs. P2. Additionally, uArg, uBCA, uIS, and uPCS express good biomarker potential when performing the comparison between subgroups, with successively increased levels from the C subgroup to the P1-P3 subgroups.

### 3.2. Correlation of Serum Metabolites with Markers of Endothelial Damage and Neurosonological Parameters

#### 3.2.1. Univariable Linear Regression Analysis

The metabolites that were most probably involved in the initiation of DKD, namely those that made a difference between the C and DKD subgroups, with a focus on the P1 subgroup, were studied first using univariable linear regression analysis. This revealed that sArg correlated negatively with the neurosonological indices (except with BHI, for which the correlation was positive) and negatively with MCP-1 and ICAM-1. In addition, sIS correlated positively with all the parameters (neurosonological and endothelial markers) except with BHI. sSorb and sBCA followed the same pattern of correlation as sIS, as can be seen in Supplementary Table S1.

#### 3.2.2. Multivariable Linear Regression Analysis

Going further, the multivariable linear regression analysis revealed that: (1) sArg, along with IMT and ICAM-1, follows a predictive model; (2) sIS correlates with RI R-ACI and ICAM-1; (3) sBCA, IMT, and MCP-1 shape a predictive model; and (4) sSorb may be a candidate marker of cerebral endothelial dysfunction by correlating with BHI and ICAM-1. These results are also expressed in Table 2.

### 3.3. The Impact of Urinary Metabolites on Renal Structures Reflected by Their Correlation through Multivariable Linear Regression Analysis, with Podocyte Damage Markers (Podocalyxin and Synaptopodin) and PT Dysfunction Markers (KIM-1, NAG)

With regard to urinary metabolites, univariable and multivariable linear regression analyses revealed that: (1) uLAC correlates positively with KIM-1 and podocalyxin, but raises multicolinearity problems; (2) a positive correlation exists between uBCA, podocalyxin and uACR; (3) uIS follows the model of uBCA by correlating positively with the same markers; and (3) uPCS associates with KIM-1 and uACR in a predictive model. Aspects regarding univariable and multivariable analyses are given in detail in Supplementary Table S2 and Table 3, respectively.

## 4. Discussion

### 4.1. Serum Biomarkers of Endothelial Damage in Early DKD

#### 4.1.1. sArg May Be a Marker of Renal and Large Cerebral Vessel Endothelial Dysfunction in Normoalbuminuric DKD

As can be seen in Table 1, sArg discriminates the healthy control group from the normoalbuminuric group and the normo- from the micro-albuminuric subgroup, respectively, with decreasing levels as DKD progresses. Table S1 and Table 2 show that sArg correlates inversely with IMT and ICAM-1 in a predictive model.

Nitric oxide synthase (NOS), which actively contributes to NO generation and reflects vascular wellness, uses Arg as a substrate, since it is a crucial precursor in energy metabolism [15]. Reduced levels of Arg may indicate endothelial dysfunction because of reduced renal synthesis, which is enhanced by arginase (ARG) activity in the urea cycle [16]. IMT predicts the development of atherosclerosis, and hence the association between IMT and Arg may indicate endothelial dysfunction in the large cerebral vessels. The association between Arg and ICAM-1 suggests the concurrent malfunctioning of renal endothelial cells. These results are in agreement with those pointed out by Oblak et al., in which endothelial dysfunction is described as highly polymorphic in different vascular beds [17].

From a clinical perspective, sArg may be used as a biomarker of incipient cerebral atherosclerosis and renal endothelial dysfunction in patients suffering from DKD in the normoalbuminuric stage. Furthermore, its measurement may be used to guide L-arginine supplementation or to assess the increased activity of ARG, which may open future perspectives of enzymatic, targeted treatments.

#### 4.1.2. sBCA Is a Biomarker of Renal and Cerebrovascular Endothelial Damage in Early DKD

Our study displays that sBCA is transitionally increasing from normo- to macroalbuminuria, differentiates C vs. P1 and P1 vs. P2, and correlates positively with IMT and MCP-1, results that are strengthened by significant *p* values.

Carnitines are involved in the intra-mitochondrial transport of fatty acids (FA), which are first converted into Acyl Coenzyme A (CoA) in the cytoplasm. Acyl CoA enters the mitochondria and undergoes multiple chemical processes. In the end, it results in the formation of Acetyl CoA, which represents the source of acetylcarnitines, under the carnitine acetyl transferase (CRAT) action, ATP, by entering into the trycarboxilic acid cycle, and cholesterol and ketone bodies [18]. In a study performed on patients with cardio-vascular disease and T2DM, it was demonstrated that in this type of patient the cleavage of FA is affected, with a subsequent over-production of acetyl CoA and acetylcarnitines. A high amount of acetyl CoA will trigger insulin resistance in a diabetic state, resulting in impaired nitic oxide mediated vasodilatation [19]. Butenoylcarnitine is a short-chain acylcarnitine discovered only in two consecutive studies conducted by Pena et al., the results of which correspond with our findings [20,21].

Thus, our study also supports the statement in which butenoylcarnitine is a promoter of renal and cerebrovascular endothelial dysfunction. Its discovery may be included in biological panels and may address the issue of DKD diagnosis precocity. A combination of pharmacological therapy with 4-(ethyl(dimethyl)ammonio) butanoate (methyl-GBB) and physical activity may reduce insulin resistance in diabetic mice, according to the intriguing results presented by Liepinish et al. [22]. From a therapeutic standpoint, short-chain acylcarnitines may therefore serve as targets and substrates for the development of the drug which might reduce their levels and delay the onset of DKD.

#### 4.1.3. sIS and Its Implication in Endothelial Dysfunction

Our results reveal successively increasing levels of sIS from the C to P3 subgroups, a positive correlation of indoxyl sulfate with markers of endothelial dysfunction (ICAM-1) and PI R-ACI, and a good differentiation between the P1 vs. C and P2 subgroups. These results are consistent with the study performed by Wang et. al. regarding the implication of indoxyl sulfate on the endothelial surface, in which it strongly correlates with ICAM-1 [23].

Indoxyl sulfate is a tryptophan derivative which follows the indole pathway, is absorbed in the gut and filtered by the glomeruli, and subsequently reabsorbed and secreted by the proximal tubule; it is extensively studied in patients suffering from chronic kidney disease (CKD). This metabolite has negative effects on the endothelial progenitor cells by inducing oxidative stress, with subsequent cell apoptosis [24]. In mature endothelial cells, indoxyl sulfate exerts its effects through the aryl hydrocarbon receptor (AhR), triggering intra-cellular production of proteins involved in inflammation and leukocyte adhesion such as ICAM-1 and MCP-1 [25]. In a study performed by Wang et al., it was pointed out that indoxyl sulfate associates with endothelial dysfunction by correlating with the vascular reactivity index in patients with advanced stages of chronic kidney disease [23]. In a study conducted on rats, Shimizu et al. managed to demonstrate that ICAM-1 is activated in renal proximal tubular cells, due to ROS production [26].

Data concerning its implication in the mechanisms that initiate vascular complications due to T2DM are sparse, especially regarding the brain endothelium. Our study points to a specific implication for indoxyl sulfate at a vascular level, especially in the cerebrovascular territory. The fact that this metabolite is correlated with RI R-ACI indicates its implication in cerebral vessel remodeling, implicit in early cerebrovascular endothelial damage in the incipient phases of DKD. The correlation of indoxyl sulfate with ICAM-1 two sides, and represents the implication of this metabolite in the mechanisms of systemic endothelial dysfunction and the targeted damage exerted on renal tubular cells by enhancing ICAM-1 expression at this level.

As sIS is a protein-bound metabolite, its clearance cannot be realized through hemodialysis; thus, the only ways to impede its effects are to: (1) decrease its gut absorption by the usage of carbon absorbents; (2) manipulate gut bacteria by the administration of probiotics, prebiotics, and symbiotics; [27] and (3) decrease its hepatic production by the administration of hepatic sulfotransferase inhibitors, as pointed out by Saito et al. [28].

#### 4.1.4. sSorb Serves as a Biomarker of BBB Damage and Decreased CVR in Early DKD

Our study showed that sSorb associates negatively with BHI and positively with ICAM-1, data presented in Table 2. In addition, these metabolite levels are dysregulated when comparing P1 vs. C and P2 subgroups. Prolonged elevated glycemia levels lead to a shift in the glycolysis process, towards the polyol pathway, with the subsequent enhanced production of sorbitol under aldose reductase activity. This then leads to an imbalance between NAPH and NADH levels, with impaired NO production [29].

Cerebral small vessel disease (CSVD) is a disorder characterized by endothelial dysfunction that may be detected via MRI or cerebral Doppler ultrasound. These methods either show cerebral lacunes, microinfarcts, microbleeds, and white matter hyperintensities, as is the case with MRI, or they reveal poor vasodilatation, through the use of complex neurosonological methods [30] Because these techniques of diagnosis fail to detect early endothelial damage, sorbitol determination may be useful in assessing BBB function and the CVR, thus serving as a future possible therapeutic target.

The clinical implication of sSorb resides in the fact that it might be a biomarker of early DKD diagnosis and early diagnosis of CSVD. Its dysregulated levels may indicate an enhanced polyol pathway activity, which may be counteracted by developing innovative enzymatic inhibitors besides the current ones, namely aldose reductase inhibitor SNK-860 [31].

### 4.2. Biomarkers of Podocyte Dysfunction and Proximal Tubule Injury Assessed by Urine Analysis

#### 4.2.1. uBCA Promotes Podocyte Injury in DKD

In this study, even though uLAC expressed a specific linear multivariable correlation with podocalyxin and KIM-1, no differences between controls and DKD subgroups were observed. Thus, we may not consider uLAC as a DKD biomarker.

In addition, our study reflected progressively increasing levels from healthy subjects to macroalbuminuric patients, expressing a differentiation between the C and DKD subgroups, but not expressing differences when comparing the P1 vs. P2 subgroups.

Enhanced levels of acylcarnitines are indicators of mitochondrial dysfunction. Furthermore, this suggests an impairment in FA oxidation, and a subsequent low energy production. Podocytes and proximal tubular cells are highly susceptible to impaired energetic substrate, which results in the intracellular accumulation of fatty acids, lipotoxicity, and cell apoptosis [32]. Butenoylcarnitine found in our patients’ urine correlated with uACR and podocalyxin. This indicates that buteonylcarnitine may be considered as a biomarker of podocyte injury in DKD.

#### 4.2.2. Uremic Toxins and Their Involvement in Podocytes and Proximal Tubule Damage

uIS correlates positively with podocalyxin and uACR, a fact which not only reflects oxidative stress in endothelial cells, but also damage at the podocyte level.

In a study conducted by Itchii et al., it was demonstrated that the long exposure of podocytes to IS determines cell damage and podocyte foot effacement, resulting in the defect of slit pore diaphragm [33]. This experiment is strengthened by our results, which show a positive correlation between uIS and podocalyxin. uIS may only be considered to be a biomarker of podocyte damage in DKD in general, but not to be of use in discriminating the normoalbuminuric subgroup from the other groups.

The levels of uPCS follow an increased trend from controls to macroalbuminuria. Our study also reveals that this metabolite discriminates controls from the P1 and P1 from the P2 subgroups. Furthermore, multivariable analysis showed that uPCS correlates positively with KIM-1 and uACR.

In a diabetic context, a shift from glucose to protein metabolism takes place. Thus, PCS is a protein-bound uremic solute that is produced in the small intestine following the phenylalanine-to-p-cresol route [34]. PCS excretion occurs by glomerular filtration and proximal tubule reabsorption through OAT receptors on the baso-lateral membrane of proximal tubular epithelial cells [35]. Watanabe et al. discovered that PCS may induce ROS production and oxidative stress in renal proximal tubular cells in chronic kidney disease [36]. Our results indicate that uPCS is a biomarker of PT injury in diabetic patients with incipient DKD, by correlating with KIM-1. Furthermore, by measuring uPCS, the diagnosis of DKD may be pointed out earlier, which is an opportunity for the clinician to promptly intervene therapeutically.

### 4.3. A Brief Overview Regarding Metabolite Clinical Impact

We have previously emphasized the importance of oral supplementation with metabolite analogues such as L-arginine, prebiotics, probiotics, and symbiotics. Additionally, the administration of specific metabolite receptor inhibitors, namely SNK-860 and hepatic sulfotransferase inhibitors, has been highlighted. However, in order to minimize bias in the interpretation of the action of these substances, prospective randomized controlled trials are required to support our assumptions. These investigations would allow for the evaluation of substance therapeutic efficacy and future clinical use.

Our study has several limitations. First, this is a pilot cross-sectional study in which the metabolites were not determined dynamically, a fact which cannot allow us to establish causality. Second, the number of enrolled patients was limited, due to the COVID-19 pandemic. Thus, the statistical power of the investigation might be interfered with by the low number of participants. Third, due to the unavailability of stool samples, the intestinal origin of the metabolites was identified based only on data found in the literature. Fourth, the discussion about therapeutical targets is only hypothetical, and needs to be confirmed by additional studies, such as randomized controlled trials.

However, our research has its strengths. To the best of our knowledge, this is the first study that focuses on the association of amino-acids, acylcarnitines, and uremic toxins with the biomarkers of endothelial, tubular, and podocyte damage, as well as with neurosonological parameters in early DKD and incipient CSVD. Also, the study provides meaningful data with regard to an earlier diagnosis of these two complications related to T2DM evolution. Furthermore, these results may enable better guidance for the development of additional therapeutical strategies in order to slow DKD and CSVD occurrence and progression.

## 5. Conclusions

In conclusion, our study highlights the fact that patients suffering from early DKD express a specific pattern of metabolites that are potentially derived from gut microbiota. Thus, sArg and sBCA are biomarkers of renal endothelial dysfunction and incipient common carotid artery atherosclerosis; sIS is a marker of renal endothelial dysfunction and incipient intern carotid artery arteriosclerosis; and sSorb is a biomarker of BBB dysfunction, and implicit in CSVD. In parallel, urine analysis reveals that uBCA is a marker of podocyte dysfunction, along with uIS in DKD, and that uPCS is a marker of proximal tubular dysfunction in the early stages of DKD.

## Figures and Tables

**Table 1:** Demographic data, clinical parameters, biological results, and neurosonological indices of healthy controls and of patients with type 2 DM divided into 3 stages: normoalbuminuria (P1), microalbuminuria (P2), and macroalbuminuria (P3).

	Healthy Subjects (C = 20)	Normoalbuminuria (P1 = 30)	Microalbuminuria (P2 = 30)	Macroalbuminuria (P3 = 30)
**Clinical Features**				
Age (years)	58.85 (7.25)	68.41 (4.98)	68.65 (4.91)	68.84 (4.98)
Male (nr,%)	12 (60%)	14 (46.66%)	13 (43.33%)	15 (50%)
Duration of DM (years)	0 ^●ↂ^	9.6 (3.99)	9.7 (3.99)	12.78 (3.35)
BMI	24.75 (4.39) ^●ↂ^	30.07 (4.54)	31.51 (4.01)	30.98 (5.28)
**Usual Biological Parameters**				
Triglycerides (mg/dL)	111.05 (20.73) ^†ↂ^	139.65 (51.1)	172.63 (93.63) ^■^	227.17 (107.55)
Cholesterol (mg/dL)	132.5 (24.62) ^†ↂ^	163.62 (54.39)	166.8 (57.7) ^■^	199.5 (48.1)
HbA1c (%)	4.98 (0.23) ^●ↂ^	5 (0.23) ^♦^	6.42 (1.29) ^♣^	7.15 (1.60)
eGFR (ml/min/1.73 m^2^)	97.93 (11.71) ^●ↂ^	90.42 (18.10) ^▲^	67.80 (5.44) ^♣^	49.53 (9.4)
uACR (mg/g)	5 (0.23) ^●ↂ^	7.38 (3.22) ^▲^	45.52 (47.08) ^♣^	319.86 (585.8)
**Markers of Endothelial Damage**				
MCP-1(pg/mL)	66.19 (11.31) ^●ↂ^	158.87 (23.09) ^▲^	219.64 (53.12) ^♣^	276.69 (54.70)
ICAM-1(ng/mL)	181.57 (13.21) ^●ↂ^	240 (24.53) ^▲^	300.63 (40.19) ^♣^	389.51 (28.55)
**Markers of Podocyte Damage**				
Podocalyxin/uCr (mg/g)	38.59 (9.02) ^●ↂ^	64.28 (7.8) ^▲^	136.31 (34.73) ^♣^	484.84 (117.17)
Synaptopodin/uCr (mg/g)	9.63 (3.7) ^●ↂ^	16.34 (5) ^▲^	26.67 (3.08) ^♣^	53.18 (36.6)
**Markers of Proximal Tubule Dysfunction**				
NAG/uCr (mg/g)	2.09 (0.73) ^●ↂ^	4.28 (4.34) ^▲^	13.11 (5.08) ^■^	16.06 (4.21)
KIM-1/uCr (mg/g)	37.76 (10.74) ^●ↂ^	77.7 (15.53) ^▲^	139.03 (16.05) ^♣^	642.1 (220.1)
**Metabolites Potentially Derived From Gut Microbiota**				
sArg (μM)	50.03 (10.02) ^†ↂ^	44 (10.18) ^♦^	38.4 (6.5)	38.91 (7.67)
sHA (μM)	24.33 (2.35) ^†⁑^	22.71 (1.24)	22.39 (1.72)	20.79 (5.1)
sIS (μM)	5.06 (0.45) ^●ↂ^	5.14 (0.47) ^♦^	6.51 (5.07) ^♣^	6.63 (0.6)
sLAC (μM)	5.52 (2.13)	5.72 (2.07)	5.47 (1.74)	5.73 (1.6)
sBCA (μM)	2.3 (0.1) ^ↂ^	2.25 (0.13) ^▲^	2.61 (0.37)	2.51 (0.55)
sSorb (μM)	2.54 (0.46) ^†ↂ^	2.25 (0.14) ^▲^	2.67 (0.3)	2.59 (0.33)
uArg/uCr (μM/μM)	5.26 (1.72)	6.08 (2.86) ^♦^	4.84 (3.22)	5.63 (2.51)
uLAC/uCr (μM/μM)	0.31 (0.13)	0.40 (0.26)	0.42 (0.31)	0.54 (0.54)
uBCA/uCr (μM/μM)	0.24 (0.11) ^●ↂ^	0.45 (0.23)	0.45 (0.32)	0.53 (0.26)
uHA/uCr (μM/μM)	52.55 (29.36)	54.66 (2.85)	57.75 (72.7)	55.62 (29.86)
uIS/uCr (μM/μM)	0.82 (0.37) ^ↂ^	2.07 (1.09)	1.99 (1.5) ^■^	2.44 (1.21)
uPCS/uCr (μM/μM)	3.39 (1.62) ^†⁑^	5.69 (5.14) ^♦^	5.65 (3.54)	7.64 (4.5)
**Neurosonological indices**				
IMT R-CCA (mm)	0.66 (0.43) ^†ↂ^	0.83 (0.11) ^▲^	1.01 (0.13) ^♣^	1.22 (0.16)
PI R-ICA	0.78 (0.12) ^●ↂ^	0.89 (0.13) ^▲^	1.09 (0.16) ^♣^	1.24 (0.14)
PI R-MCA	0.62 (0.7) ^†ↂ^	0.78 (0.14) ^▲^	0.97 (0.16) ^♣^	1.11 (0.14)
RI R-ICA	0.58 (0.97) ^†ↂ^	0.72 (0.07) ^▲^	0.94 (0.13) ^♣^	1.18 (0.19)
RI R-MCA	0.54 (0.36) ^†ↂ^	0.65 (0.07) ^▲^	0.97 (0.11) ^♣^	1.2 (0.1)
BHI	1.12 (0.12) ^†ↂ^	0.84 (0.11) ^▲^	0.55 (0.1) ^♣^	0.43 (0.06)

Data presented as means ± SD. *p* value was based on: Student’s *t*-test, chi-squared test, Mann–Whitney test; significance of C vs. P1: ● *p* < 0.001; † *p* > 0.001 and *p* < 0.05; significance of P1 vs. P2: ▲ *p* < 0.001; ♦ *p* > 0.001 and *p* < 0.05; significance of P2 vs. P3: ♣ *p* < 0.001; ■ *p* > 0.001 and *p* < 0.05; significance of C vs. P1 vs. P2 vs. P3: ↂ *p* < 0.001; ⁑ *p* > 0.001 and *p*< 0.05; BMI: body mass index; BHI: breath-holding index; DM: diabetes mellitus; eGFR: estimated glomerular filtration rate; ICAM-1: intracellular adhesion molecule-1; IMT R-CCA: intima-media thickness in right common carotid artery; uACR: urinary albumin/creatinine ratio; KIM-1/creat: urinary kidney injury molecule-1/creatinine ratio; MCP-1: monocyte chemoattractant protein-1; NAG/creat: N-acetyl-β-(D)-glucosaminidase/creatinine ratio; Podocalyxin/uCr: Podocalyxin-to-creatinine ratio; PI R-ICA: pulsatility index in right internal carotid artery; PI R-MCA: pulsatility index in the right middle carotid artery; RI R-ICA: resistance index in the right internal carotid artery; RI R-MCA: resistance index in the middle carotid artery; Synaptopodin/uCr: Synaptopodin-to-creatinine ratio; sArg: serum arginine; sHA: serum hippuric acid; sIS: serum indoxyl sulfate; sLAC: serum L-acetylcarnitine; sBCA: serum butenoylcarnitine; sSorb: serum sorbitol; HbA1C: glycated hemoglobin; uArg/uCr: urinary arginine-to-creatinine ratio; uHA/uCr: urinary hippuric acid-to-creatinine ratio; uIS/uCr: urinary indoxyl sulfate-to-creatinine ratio; uLAC/uCr: urinary L-acetylcarnitine; uBCA/uCr: urinary butenoylcarnitine-to-creatinine ratio; uPCS/uCr: urinary p-cresyl sulfate-to-creatinine ratio.

**Table 2:** Multivariable linear regression analysis of serum samples.

Dependent Variable	Independent Variables	Coef β	*p*	95% CI	Prob > F	R^2^
**sArg**	IMT	13.83	0.022	2.03 to 25.63	<0.0001	0.230
	ICAM-1	−0.87	<0.0001	−0.12 to −0.05		
**sIS**	RI R-ACI	−4.99	0.008	−8.64 to −1.34	<0.0001	0.179
	ICAM-1	0.03	<0.0001	0.01 to 0.03		
**sBCA**	IMT	−0.72	0.001	−1.13 to −0.32	<0.0001	0.355
	MCP-1	0.004	<0.0001	0.003 to 0.005		
**sSorb**	BHI	0.67	0.001	0.28 to 1.06	<0.0001	0.342
	ICAM-1	0.005	<0.0001	0.004 to 0.007		
	eGFR	0.01	0.002	0.004 to 0.017		

**Table 3:** Multivariable linear regression analysis of urine metabolites.

Dependent Variable	Independent Variables	Coef β	*p*	95% CI	Prob > F	R^2^
**uLAC**	Podocalyxin	−0.0005	0.011	−0.001 to −0.0001	<0.0001	0.632
	KIM-1	−0.0006	<0.0001	−0.0009 to −0.0003		
	uACR	−0.0007	<0.0001	0.0006 to 0.0009		
**sIS**	Podocalyxin	−0.002	0.008	−0.003 to −0.0005	<0.0001	0.327
	uACR	0.001	<0.000	0.001 to 0.002		
**uBCA**	Podocalyxin	−0.0005	0.002	−0.0008 to −0.0002	<0.0001	0.339
	uACR	0.0003	<0.0001	0.0002 to 0.0005		
**uPCS**	KIM-1	−0.009	<0.0001	−0.014 to −0.006	<0.0001	0.507
	uACR	0.008	<0.0001	0.007 to 0.01		

## Data Availability

The data that support the findings of this study are available from the corresponding author upon reasonable request. The data are not publicly available due to their containing information that could compromise the privacy of research participants.

## Supplementary Materials

The following supporting information can be downloaded at: https://www.mdpi.com/article/10.3390/metabo13080893/s1, Supplementary Table S1: Serum Univariable Analysis; Supplementary Table S2: Urine Univariable Analysis.
