# Supplementary material for: Metabolites Potentially Derived from Gut Microbiota Associated with Podocyte, Proximal Tubule, and Renal and Cerebrovascular Endothelial Damage in Early Diabetic Kidney Disease in T2DM Patients
Source: Metabolites. 2023 Jul 28;13(8):893. doi: 10.3390/metabo13080893 (PMC10456401; doi:10.3390/metabo13080893)
Supplement: Supplementary file 1 [file metabolites-13-00893-s001.zip › metabolites-2510220-supplementary.pdf]

Table S1 Serum univariable analysis

Dependent variable	Independent variable	R^2	Coef β	P value (prob > F)
**sArg**	IMT	0.0594	-10.49	0.0061
	PI R-ACI	0.0594	-11.15	0.0061
	PI R-ACM	0.0743	-12.09	0.0024
	RI R-ACI	0.1526	-14.06	<0.0001
	RI R-ACM	0.1216	-12.51	0.0001
	BHI	0.163	14.46	<0.0001
	MCP-1	0.1945	-0.05	<0.0001
	ICAM-1	0.1986	-0.05	<0.0001
**sHA**	IMT	0.101	-4.54	0.0004
	PI R-ACI	0.0479	-3.47	0.0127
	PI R-ACM	0.047	-3.401	0.0130
	RI R-ACI	0.0126	-1.837	0.1257
	RI R-ACM	0.0931	-3.61	0.0013
	BHI	0.0918	3.7	0.0008
	MCP-1	-0.0089	-0.0008	0.8208
	ICAM-1	0.0059	-0.004	0.2031
**sIS (μM)**	IMT	0.0555	2.947497	0.0078
	PI R-ACI	0.0316	2.490465	0.0358
	PI R-ACM	0.0442	2.556245	0.0282
	RI R-ACI	0.0331	2.164511	0.0324
	RI R-ACM	0.0431	2.291736	0.0172
	BHI	0.0572	-2.60003	0.0007
	MCP-1	0.0949	.0104853	0.0007
	ICAM-1	0.1309	.0128135	0.0001
**sLAC (μM)**	IMT	-0.0071	0.37	0.6234
	PI R-ACI	-0.004	0.603	0.452
	PI R-ACM	-0.0062	0.45	0.5628
	RI R-ACI	0.0156	1.112051	0.1029
	RI R-ACM	-0.0086	0.1820748	0.7806
	BHI	-0.0078	-0.2652648	0.6868
	MCP-1	0.0964	0.007086	0.0006
	ICAM-1	0.0952	0.0074211	0.0006
**sBCA (μM)**	IMT	0.0608	0.4235481	0.0056
	PI R-ACI	0.0798	0.5077507	0.0017
	PI R-ACM	0.0845	0.5119193	0.0013
	RI R-ACI	0.1560	0.5756828	<0.0001
	RI R-ACM	0.0875	0.4301584	0.0010
	BHI	0.0874	-0.4329471	0.0011
	MCP-1	0.2855	0.0024359	<0.0001
	ICAM-1	0.2331	0.0023264	<0.0001
**sSorb (μM)**	IMT	0.0560	0.35	0.0132
	PI R-ACI	0.0710	0.44	0.0029

	PI R-ACM	0.0593	0.403	0.0061
	RI R-ACI	0.1213	0.47	0.0002
	RI R-ACM	0.0875	0.37	0.0018
	BHI	0.0357	-0.27	0.0275
	MCP-1	0.179	0.001	<0.0001
	ICAM-1	0.187	0.002	<0.0001

Table S2 Univariable urine analysis

Dependent variable	Independent variable	R^2	Coef β	P value
**uArg**	Synaptopodin	0.0418	0.0242024	0.0186
	Podocalyxin	-0.0082	0.0004799	0.7258
	KIM-1	-0.0070	0.0004832	0.6159
	NAG	-0.0076	-0.0160105	0.6697
	RAC	0.1212	0.0017062	0.0001
**uLAC**	Synaptopodin	0.2446	0.0071333	<0.0001
	Podocalyxin	0.0492	0.0004501	0.0116
	KIM-1	0.0306	0.0002619	0.0380
	NAG	0.0231	0.00919	0.0622
	RAC	0.4088	0.000404	<0.0001
**uBCA**	Synaptopodin	0.1778	0.0216489	<0.0001
	Podocalyxin	0.0737	0.0018953	0.0025
	KIM-1	0.0742	0.0013386	0.0024
	NAG	0.0630	0.0485265	0.0049
	RAC	0.2874	0.0012031	<0.0001
**uHA**	Synaptopodin	0.0247	0.3203106	0.0559
	Podocalyxin	-0.0093	0.002758385	0.9991
	KIM-1	-0.0090	0.0031451	0.8406
	NAG	0.2038916	0.2038916	0.7378
	RAC	0.0626	0.0205445	0.0050
**uIS**	Synaptopodin	0.1778	0.0216489	<0.0001
	Podocalyxin	0.0737	0.0018953	0.0025
	KIM-1	0.0742	0.0013386	0.0024
	NAG	0.0630	0.0485265	0.0049
	RAC	0.2874	0.0012031	<0.0001
**uPCS**	Synaptopodin	0.2352	0.0948555	<0.0001
	Podocalyxin	0.0713	0.0071605	0.0029
	KIM-1	0.0496	0.0043104	0.0113
	NAG	0.0451	0.1614279	0.0150
	RAC	0.3808	0.0052878	<0.0001
